# Implementing and evaluating care and support planning: a qualitative study of health professionals’ experiences in public polyclinics in Singapore

**DOI:** 10.1186/s12875-023-02168-5

**Published:** 2023-10-19

**Authors:** Vikki A. Entwistle, Sharon McCann, Victor Weng Keong Loh, E Shyong Tai, Wee Hian Tan, Tong Wei Yew

**Affiliations:** 1 Health Services Research Unit, Health Sciences Building, University of Aberdeen, Foresterhill, Aberdeen, AB25 2ZD Scotland, UK; 2 School of Divinity, History, Philosophy and Art History, University of Aberdeen, Aberdeen, Scotland; 3 Centre for Biomedical Ethics, National University of Singapore, Singapore, Singapore; 4 Division of Family Medicine, Yong Loo Lin School of Medicine, National University of Singapore, Singapore, Singapore; 5 Department of Family Medicine, National University Health System, Singapore, Singapore; 6 Department of Medicine, National University Hospital, Singapore, Singapore; 7 Department of Medicine, Yong Loo Lin School of Medicine, National University of Singapore, Singapore, Singapore; 8 National University Polyclinics, National University Health System, Singapore, Singapore

**Keywords:** Self-management support, Care and support planning, Diabetes, Professional-patient relations, Person-centred care, Qualitative interviews, Professional education, Continuity of care, Healthcare improvement

## Abstract

**Background:**

Two polyclinics in Singapore modified systems and trained health professionals to provide person-centred Care and Support Planning (CSP) for people with diabetes within a clinical trial. We aimed to investigate health professionals’ perspectives on CSP to inform future developments.

**Methods:**

Qualitative research including 23 semi-structured interviews with 13 health professionals and 3 co-ordinators. Interpretive analysis, including considerations of how different understandings, enactments, experiences and evaluative judgements of CSP clustered across health professionals, and potential causal links between them.

**Results:**

Both polyclinic teams introduced CSP and sustained it through COVID-19 disruptions. The first examples health professionals gave of CSP ‘going well’ all involved patients who came prepared, motivated and able to modify behaviours to improve their biomedical markers, but health professionals also said that they only occasionally saw such patients in practice. Health professionals’ accounts of how they conducted CSP conversations varied: some interpretations and reported enactments were less clearly aligned with the developers’ person-centred aspirations than others. Health professionals brought different communication skill repertoires to their encounters and responded variably to challenges to CSP that arose from: the linguistic and educational diversity of patients in this polyclinic context; the cultural shift that CSP involved; workload pressures; organisational factors that limited relational and informational continuity of care; and policies promoting biomedical measures as key indicators of healthcare quality. While all participants saw potential in CSP, they differed in the extent to which they recognised relational and experiential benefits of CSP (beyond biomedical benefits), and their recommendations for continuing its use beyond the clinical trial were contingent on several considerations. Our analysis shows how narrower and broader interpretive emphases and initial skill repertoires can interact with situational challenges and respectively constrain or extend health professionals’ ability to refine their skills with experiential learning, reduce or enhance the potential benefits of CSP, and erode or strengthen motivation to use CSP.

**Conclusion:**

Health professionals’ interpretations of CSP, along with their communication skills, interact in complex ways with other features of healthcare systems and diverse patient-circumstance scenarios. They warrant careful attention in efforts to implement and evaluate person-centred support for people with long-term conditions.

**Supplementary Information:**

The online version contains supplementary material available at 10.1186/s12875-023-02168-5.

## Background

Over recent decades, significant efforts have been made internationally to ensure that health services (and especially primary care) support the growing numbers of people with chronic diseases to manage these effectively in the context of their daily lives [1–3]. Increasing emphasis is also placed on the idea that such support should be ‘person-centred’ and oriented to enable people to live (and die) well with their long-term conditions [4, 5]. In part this reflects recognition that much of the value of support for self-management lies in its potential to improve the social and psychological aspects of life with long-term conditions. These aspects not only can significantly impact how well people manage those conditions, but also are important in their own right. At a population level, the effects of interventions on biomedical markers might be modest, especially over the short term [6], but respectful, responsive support can improve wellbeing more broadly and may mediate some biomedical improvement (or at least limit deterioration) over the longer-term [6–9].

The concept of person-centredness is variously explained and interpreted but includes notions of health professionals focusing on the person not just the disease, working respectfully *with* the person, enabling their agency, orienting to address the person’s own concerns and priorities for their wellbeing, and recognising the practical realities of their lives [5, 10]. The latter may be particularly important when poverty and social inequality constrain people’s scope to improve their health via lifestyle ‘choices’ [11, 12].

The implementation of person-centred support for self-management requires widespread adoption of person-centred values (including by institutional leadership); professional acquisition of relevant skills (including microskills of communication); and development of organisational structures (including information systems) to underpin delivery, including by preparing patients [9, 13].

One of the most established approaches to person-centred support for self-management, especially in the UK, is personalised Care and Support Planning (CSP) [13]. As developed by Year of Care Partnerships® (an NHS organisation dedicated to the implementation of personalised approaches to care), this approach emphasises the importance of a meaningful conversation between a prepared patient and a CSP-trained health professional. To help patients prepare for their CSP conversation appointment, they are sent a care planning letter in advance. This letter prompts them to consider any issues that they would like to discuss with the doctor or nurse. It summarises their biomedical test results (including past trends) before asking them to consider what they would like to work on this year. Health professionals are trained to actively listen to the patient’s concerns and perspectives on what is important before sharing any additional thoughts from their professional perspective, then collaboratively supporting the patient to prioritise and develop specific goals relating to what matters to them, and to formulate a realistic plan for achieving them [13].

In Singapore, CSP was first introduced for patients being treated for diabetes in a hospital setting in 2017. The endocrinologists involved recognised that the approach could also be appropriate for primary care, including within polyclinics. Polyclinics are multi-professional public sector organisations that (separately from private sector family doctors) provide a broad range of primary healthcare services at subsidised prices for many of the rising numbers of people with diabetes [9, 14, 15]. Singapore’s Ministry of Health funded a three-year trial of CSP using materials and processes adapted from the Year of Care Partnerships programme to suit the local context. The trial, Patient Activation through Community Empowerment/Engagement for Diabetes management (PACE-D), is set in four polyclinics. Two polyclinics each developed their systems and introduced CSP into the working arrangements of one or two professional teamlets (small groups of doctors and nurses that provide care for a panel of patients) [16, 17]. The PACE-D trial aims to recruit all adults with diabetes who can communicate in English, Chinese or Malay. The primary outcome is HbA1c levels (which the research team acknowledges fails to capture many potential benefits of CSP) [16].

We report here on a qualitative study of health professionals’ experiences of CSP, conducted to complement both the PACE-D trial and an associated study of patients’ experiences. We aimed to investigate professional perspectives on the introduction of CSP with a view to informing any more widespread adoption of CSP in Singapore. We were particularly aware, from research in UK and Australian settings, that health professionals can vary in their interpretations (including value emphases) and enactments of person-centred support for self-management [18–20] and can experience tensions when attempting to adopt such approaches [21, 22], so we were also interested in the potential of this study to further illuminate these issues, and in a South-East Asian context. Our findings and analysis have implications for the implementation and evaluation of CSP and similar approaches internationally.

## Methods

This was a qualitative, interpretive study. We conducted individual interviews with health professionals working directly with patients for CSP. For context, we also observed the training session for doctors and nurses involved in PACE-D and team huddles convened to support CSP implementation.

### Individual interviews

All doctors and nurses who conducted CSP conversations for PACE-D were invited to take part, as were PACE-D coordinators (a new teamlet role introduced to help recruit patients, arrange appointments, and collect data for the trial). Eligible staff were identified and approached by a PACE-D administrator and subsequently a PACE-D coordinator who also helped arrange interviews. Informed consent was documented before interviews commenced.

Participants were told that the study aimed to learn from their perspectives, including to identify any concerns or difficulties with CSP, to complement and inform the interpretation of the PACE-D trial data when it became available, and to inform decisions about whether and how CSP would continue beyond the trial. The interviewer (VAE) was introduced to health professionals during their CSP training and first huddles as a non-clinical Professor, new to Singapore, with an interest in person-centred care and self-management support.

We planned to conduct two face-to-face interviews with each health professional: one relatively soon after CSP conversations were initiated and a second at least 9 months later once they had more experience with the approach and had seen some patients for follow-up appointments. This reflected recognition that skills can develop with practice and that experience and perspectives can change with time. We were mindful that when interventions such as CSP are introduced, the health professionals who are expected to adopt new practices might benefit from some kind of support as they get used to them, and we thought that conducting two interviews rather than just one, when practices had become more settled, could be useful. In practice, first interviews started later than originally envisaged due to an initial delay in recruiting patients to the study and not all were completed before COVID-19 resulted in access restrictions. Further delays arose when VAE relocated, necessitating a revision to the study protocol and the securing of ethics committee approval to conduct interviews online and from overseas. Some health professionals’ first interviews thus did not take place until at least 12 months after CSP conversations were commenced, and in these circumstances, we judged a second interview unnecessary. To avoid confusion, we refer to the interviews as ‘wave 1’ (conducted relatively soon after CSP was implemented) and ‘wave 2’ (conducted at least 9 months after CSP was implemented). These are indicated as w1 or w2 after quotations. For health professionals who were interviewed twice, the minimum time between first and second interviews was 9 months (average 11 months). We note that the CSP experience levels of health professionals at the time of each wave varied because of a combination of leave and secondment arrangements and, to some extent, preferences for conducting CSP conversations.

The interviews were conversational in style, supported by a topic guide that (for doctors and nurses) prompted coverage of: their role in relation to PACE-D, how they had been introduced to CSP and what they had first thought of it; examples of occasions when they thought CSP conversations had gone more and less well; how CSP conversations went compared with previous consultations for diabetes; perceived advantages and disadvantages for patients and for health professionals; any challenges in delivering CSP well; and whether and why they would or would not be inclined to continue with CSP beyond the PACE-D study, or to recommend it to other polyclinics. The topic guide was modified for interviews with PACE-D coordinators and developed over time to reflect questions of interest identified from previous interviews (for example, whether and how CSP works differently for different patient groups). It was annotated for second interviews to pick up points raised previously by each participant, including to check whether and how experiences or views had changed.

Interview recordings were a mean of 48 minutes long (range 36 to 74 minutes).

Audio-recordings of the interviews were transcribed by medical students on a student work scheme. VAE checked and corrected the transcripts against the audio recordings.

### Observation of training session and huddles

By invitation of PACE-D study leads, and with prior agreement from trainers, VAE was introduced to participating health professionals, as noted above, as one of several observers of the local CSP training session for health professionals and of the first lunchtime huddle sessions convened by the trainers to give the health professionals an opportunity to discuss how CSP was going and share concerns or practical tips. Participants were told that the observation could help provide background understanding for the interview study and help inform interpretation of PACE-D trial findings and decisions about the use of CSP beyond the trial. We explained that we were not analysing who said what in the training sessions or huddles, and that no quotations would be used. VAE interacted conversationally with health professionals and trainers during refreshment breaks and was included in lunch arrangements for the huddle. She also participated occasionally, for example, stepping in to make up a pair and play the role of a patient for a role play exercise during the training session, briefly confirming during an early huddle that health professionals in the UK also reported variation in how much preparatory thinking patients did ahead of CSP conversations, and (more significantly) presenting the preliminary analysis from the interview study for discussion at the final huddle observed.

A comprehensive set of slides and documents was provided for reference from the training. Note-taking from the huddles was handwritten, not formally structured, and brief. It focused on summarising topics of concern and key features of meetings (e.g. started late because clinics overran; some participants visibly very tired). Huddles were scheduled for an hour, but typically started late. In total VAE attended 7 of 11 huddles and watched recordings of 2 huddles that she could not attend in person. This amounted to approximately 6 hours of active meeting time observed.

The observations helped to inform interview questions (for example, prompting inquiry about how CSP conversations were documented in medical records) and provided context to support interpretive analysis of interview data (particularly confirming the nature and strength of concern about logistical challenges, including those associated with long patient queues in clinic).

We decided not to observe CSP consultations, both to avoid additional intervention in the context of the PACE-D trial and because the health professionals were already under significant pressure.

### Analysis

Our qualitative analysis was broadly interpretive. It was practically oriented, but with awareness that theoretical development could also be practically useful. Primarily, we sought to complement and support interpretation of the PACE-D trial results, and to inform any plans to extend CSP beyond the trial period and initial polyclinic sites. To this end, we were sensitised by key ideas from Normalization Process Theory (including the relevance for implementation of actors’ views of intervention components, coherence with existing ideas and systems, cognitive participation, collective action and reflective monitoring) [23], although we did not use this formally as an analytic framework. An additional interest, as noted in Background, was in any variations in health professionals’ interpretations and enactments of CSP, and the potential significance of those for implementing and evaluating person-centred approaches to support.

We deployed multiple analytic strategies to develop our understanding of what was going on in this complex and dynamic situation. At a first, we might say surface level of analysis, we focused on what health professionals told us directly, and looked for similarities and differences in what they said, for example about what they saw as the main differences between CSP conversations and their previous approaches to annual review and “usual” consultations, the challenges they experienced with delivering CSP well, and whether they thought CSP should continue beyond the PACE-D clinical trial. We also compared health professionals’ self-reported interpretations and enactments of CSP with the person-centred principles, communication skills and goal-setting action planning strategies covered in the CSP training and envisaged in the broader literature. At another, we might say higher level of analysis, we considered how (sometimes subtly) different interpretations, enactments, experiences and evaluations of CSP were connected, attending not just to health professionals’ own reflections but also looking at how particular interpretations, enactments, experiences and evaluative judgements clustered within and varied across health professionals’ interviews.

In terms of tools used or processes followed for these analyses, after close reading and manual note-making on the early (wave 1) transcripts, VAE developed a ‘broad-brush’ set of codes to help organise the data. This reflected the research questions and interview structure as well as points of interest discussed with other authors. SM subsequently worked line by line through all the transcripts using NVivo software to apply these codes and collate data for further review. This ‘chunking’ allowed us to consider all responses to specific interview questions (for example inviting a description of a CSP conversation that had gone well, and asking for comment on how they found the training and huddles) and to bring together what was said about particular CSP processes (for example the care planning letter, or the goal-setting and action planning stage of the consultation) and more obviously evaluative judgements. It was particularly useful for supporting identification of the range of health professionals’ interpretations, enactments, experiences (including of challenges) and views about the purpose and success or otherwise of CSP. We also used it to start to identify potential shortfalls from CSP as introduced in training, coding data as relating to this either if health professionals themselves mentioned concerns or if researchers identified potential discrepancies on the basis of health professionals’ descriptions of what they did.

To support consideration of potential links between what each health professional said directly about the purpose(s) and value of CSP, their accounts of practical examples, the challenges they reported, and their evaluative reflections, we constructed analytic charts of a kind associated with a framework approach [24], although with relatively broad headings and thus relatively long cell entries. These were constructed on MS Word. VAE worked again through all the interview transcripts, reading each carefully in their entirety and summarising directly from transcript to chart row for each participant: evidence of their emphasis in interpretation and overall evaluation of CSP; the way they described challenges to CSP and their enacted responses to those; their accounts of working with patients with different characteristics, and connections drawn by the health professionals or researchers between these. Information from wave 1 and wave 2 interviews was recorded in different coloured text. An additional step of further analytic reflection, annotation of chart printouts and distillation from initial ‘long-version’ into ‘shorter’ summary charts helped develop an explanatory account of how what we loosely characterised as narrower and broader views about the purpose(s) of CSP, and narrower and broader initial skill repertoires, could interact with practical-situational challenges in the polyclinic to shape health professionals’ enactments, personal experiences and evaluative judgements of CSP. This analysis was credibility checked and clarified in discussion with all authors. Although it cannot be fully demonstrated via direct excerpts from interview transcripts alone, we developed summaries of four health professionals’ interpretations, enactments and evaluations of CSP to illustrate how these cluster (see Table 1 in Results below).

**Table 1:** Illustration of contrasts between health professionals’ interpretations, enactments and evaluations of CSP^a^

**HP Eng**	**HP Chen**	**HP Ho**	**HP Wu**
HP Eng gave a limited account of the purpose and ethos of CSP: “let patient talk a bit more… so that we can understand the patient more” and so “help them manage their chronic condition better than the usual approach”. He saw CSP as “quite similar” to usual care but with less reliance on medication and more attention to diet and exercise. In both interviews, HP Eng referred to CSP as “patient-centric”. In w1 he explained this meant “patients realize that all this is for their own good” and that “they also had to take care of their own health”. In w2, he linked the concept to listening to patients’ concerns and giving advice relevant to their situation. HP Eng valued the graphs in the CSP letter as making it “easier for [patients] to understand” “the target we are aiming for”. He said in w1 “one of the things I’m hearing about the idea of the CSP is perhaps that it’s the patient’s ideas about what they can do” but had no examples of this. In w2 he more confidently described going through each indicator in the letter, using the graphs to give patients “a clearer picture” and provide a focus for goal setting. HP Eng routinely asked patients about their “diet, exercise and then whether they comply with the medication, any side effect or not”. HP Eng thought the few patients he had seen by w1 were “not that interested”, because they had not completed the results letter. At w2, he still found most patients “not interested”, perhaps because they had financial worries with which “we can’t help the patient”. HP Eng thought CSP could help him understand a patient’s situation and so tailor advice-giving. However, it was “not so helpful” if patients “don’t give you much” or “are not so willing to work with you”. HP Eng saw no impact of CSP on empathy, rapport or respect in his communication with patients. He said he rushed CSP conversations because “the queue builds up and we can’t afford” 30 minutes. HP Eng saw it as a limitation of CSP that patients were often not motivated to modify their diet or exercise to improve diabetes control. HP Eng did not directly answer questions about continuing with CSP but emphasised that it was difficult given clinic time pressures.	HP Chen regarded improvement of diabetes control, and especially HbA1c, as the purpose of PACE-D and focus of the CSP conversation. He thought CSP could be “quite helpful” for patients with poorly controlled diabetes who come prepared and motivated. However, he “rarely” saw such prepared and motivated patients, especially through to follow-up. At w1, HP Chen had not seen any clear examples of improvement in HbA1c. By w2, he had seen one or two patients “that really achieve their goal” of improved sugars or weight. HP Chen found CSP “tough” and “tiring” in the busy polyclinic with the stress of the patient queue and the challenge of changing pace between usual and PACE-D consultations. This did not improve with experience. HP Chen admitted “cut[ting] patients short” in the telling of their concerns, finding mentions of non-life-threatening problems an unhelpful diversion. He sometimes skipped to goal setting. When reviewing CSP letters with patients, HP Chen prioritised blood sugar, blood pressure and cholesterol levels. When the patient queue allowed, he tried to “pause a little” to ask patients about family, work and what is important to them before sharing “some sad stories” about patients with uncontrolled diabetes who didn’t listen to advice and suffered complications. Although some patients would “just keep quiet” he could “try to push” those “able” to say “of course they wanted to be still independent” to help them set goals and achieve targets for sugar control. If time allowed, HP Chen would ask about their routine so his advice could be more practical for their situation. HP Chen thought patients could feel more respected when given time to share their concerns but saw the time it took to hear these as a disadvantage for staff. HP Chen favoured the faster resolution and lower resource use of usual consultations but thought neither these nor CSP made much difference for patients with poorly controlled diabetes who did not take prescribed medicines. For HP Chen, the main challenges of CSP were time constraints and patients’ lack of motivation. With uncertainty about CSP’s impact on diabetes control and concern about staffing levels and resources, HP Chen was not inclined to recommend continuing CSP for all people with diabetes but perhaps not averse to continuing with a more select patient group.	HP Ho talked very positively about CSP training as having “opened up” for him a new way of consulting. He saw CSP as an approach that could help him “understand the patients better”, work “together” with them “toward a common ground” and so improve their care. It took time for HP Ho to get beyond consciously telling himself to “change mode” for CSP and get comfortable using the approach. He also needed to remind himself about CSP after time away from the Teamlet. His self-reminding focused primarily on “shared decision-making” the “OARS” skills and “patients’ goals”. HP Ho found some patients were reluctant to say much, perhaps because they were not used to the CSP approach. However, he could usually “fish out” something useful in a conversation and valued coming to understand the patient even a bit better. With CSP, HP Ho heard things that he didn’t used to hear, including what people were concerned about and why. If he learned about a difficult home or work situation, he could at least show empathy and not suggest or expect impossible diet or exercise changes. HP Ho used CSP type questions in other consultations as well as designated CSP conversations. His reflections on goal setting with patients who found this difficult led him to suggest that sometimes “starting” something might be an appropriate goal. HP Ho thought CSP gave health professionals a better chance of improving healthcare and outcomes (he had a keen interest in reducing cardiovascular risk). CSP could help him increase patients’ motivation – for example supporting someone to lose weight because they wanted to feel better about themselves. He also appreciated that CSP could ensure patients felt more listened to and respected. Even if the agreed action was only to keep medications at the same dose, he could be more confident that was what the patient wanted. HP Ho was not sure that CSP had benefitted all the patients he saw (he lacked evidence, including about patient satisfaction) but he was confident it benefited some. He would “strongly encourage” continued use of CSP and the incorporation of its principles into daily consults. He stressed that the CSP system depended on the ongoing support of PACE-D co-ordinators and extended consultation times.	HP Wu stressed the collaborative aspects of CSP. It “made a lot of sense” to her that patients got their lab results before consultations, and she valued how CSP “flipped” the conversation to ask what was important to the patient. HP Wu had started using what she learned from CSP training immediately and found patients responded positively to open questions about what they thought. She now finds it “difficult to not” use this approach in her consultations. HP Wu emphasised a need for the collaborative ethos of CSP to be followed through between the main CSP conversations. She prepared carefully for CSP conversations and follow-ups and had found a way to search medical records for panels of lab tests to help find notes from the CSPs that followed. HP Wu admitted struggling with long patient stories that she thought she could predict the end of. “Speed is a thing” in the polyclinic and professional training had taught her that good doctors diagnose and act quickly – but she had learned to listen and see where the story took her. HP Wu gave detailed examples of different kinds of benefit derived from hearing what a patient would have been less able to share in usual consultations HP Wu appreciated that various forms of progress might be made in a CSP conversation even if no goals were set or actions plans made. She stressed a need to avoid imposing a tick-box process orthodoxy for CSP on health professionals: sometimes, for example, goal setting and action planning was less necessary or appropriate. HP Wu also reflected that as a deeply personal process, goal-setting could make patients feel vulnerable so needed to be supported carefully. HP Wu considered CSP beneficial for both patients and health professionals. Even at its worse a CSP conversation would be no worse than a usual consultation for patients. She favoured continuing with the approach but noted that while some health professionals spent time on CSP with some patients, others would be working with a queue of patients who would receive less contact time.

^a^We have changed the gender pronouns used for some participants to protect identities

Observations and documentation from the training, together with publications about CSP, were used primarily as a reference point against which health professionals’ accounts and reported enactments of CSPs were considered. Observations of the huddles were used primarily to summarise how these featured in the implementation process, but also reinforced what health professionals shared in individual interviews about organisational and logistic challenges of CSP, and allowed us to see that further input from CSP trainers was consistent with the original training in terms of the person-centred principles it emphasised and communication skills it sought to bolster.

A summary of the analysis reported here was shared with the PACE-D leads and presented to participants and others during a PACE-D huddle session, providing an opportunity for discussion and further comment and refinement before this manuscript was finalised.

In the Results and associated Tables, the names attached to quotations are all pseudonyms. To preserve participants’ confidentiality, we have not distinguished between doctors and nurses, we have sometimes changed the gender of pronouns, and we have not provided polyclinic affiliations. Quotations are verbatim but we have deleted hesitation sounds and spoken word repetitions. Where we have omitted words to improve clarity, this is indicated by ‘…’. Where we have added or condensed explanatory points they are within [ ]. Some Singlish constructions may look grammatically odd to readers more accustomed to British English, but the meaning should be clear. The expression “Lah” serves to add emphasis to a point.

## Results

We interviewed 11 doctors, 2 nurses and 3 PACE-D coordinators. 7 doctors and 1 nurse were interviewed twice and 2 PACE-D coordinators asked to be interviewed together, so there were 23 interviews in total. 14 interviews (all in the second wave) were conducted online.

Our sample includes all but 2 of the health professionals trained to conduct CSP conversations within the PACE-D trial: 2 eligible nurses declined interviews because they conducted very few CSP conversations. 11 participants were from one polyclinic and 5 from the other, reflecting different staffing arrangements.

The interviews and huddle observations confirmed reports from PACE-D leaders that both polyclinics managed to establish a CSP system for people with diabetes, and to sustain this through the substantial disruptions of COVID-19 and introduction of a Next Generation Electronic Medical Record system. (At the time of writing CSP has been provided in these polyclinics for over three years – continuing beyond the trial). They also highlighted that the PACE-D coordinators came to play key roles in keeping key CSP processes going (not just supporting research components).

We report our findings in three main sections. First, we outline the understandings of CSP evident in doctors’ and nurses’ accounts, including the key contrasts they saw between CSP and their previous ways of working, and the range of benefits they attributed to CSP. Important variations in health professionals’ enactments of CSP are introduced within this section.

Second, we consider the challenges to CSP that participants reported as arising from (a) the situated characteristics of local patients and (b) organisational factors and policy pressures. This section also notes variations in health professionals’ interpretations of and enacted responses to these challenges. It indicates how both the external challenges and variations in health professionals’ enactments can impact the achievement and recognition of potential benefits of CSP.

Third, we examine health professionals’ overall evaluations of CSP and their inclination to extend its use beyond PACE-D. We show that these evaluations are somewhat contingent on health professionals’ interpretive emphases, experiences of challenges, and awareness of variations in their collective enactments.

In the Discussion, we develop our analysis of how variations in health professionals’ interpretive emphases and skill repertoires impact their enactments of CSP, scope to improve these with experience, evaluative judgements of CSP and motivation to continue with the approach.

The findings and analysis were endorsed by participants when presented to their team huddle. Health professionals particularly appreciated recognition of the challenges they faced and the recommendations for future organisational support for CSP.

### Understandings and reported enactments of CSP

#### When CSP goes well: ‘ideal’ patient behaviours and outcomes

When asked early in their interviews for an example in which CSP went relatively well, participants gave strikingly similar responses. In both waves, all included most of the following elements: a patient comes to their CSP conversation actively ‘prepared’ (having read and reflected on their care planning letter), engages fairly readily in discussion, is willing to act to improve their health, proposes or works with the health professional to set relevant goals and a realistic action plan, leaves the CSP conversation motivated, sticks to their plan and sees (or would see) improvement in targeted biomedical markers at follow-up. A selection of these ‘good example’ accounts is provided in Additional Table 1.

In practice, of course, CSP did not always work along these lines. Participants consistently reported that only a minority of patients came to CSP appointments clearly familiar with their test results and able and willing to discuss ideas about their health, let alone set and stick to plans for health-related behaviour change that positively impacted their biomedical markers. Some participants seemed surprised by this and there was some variability in how and to what extent they became reconciled to it.

We will return shortly to consider variations in patients’ engagement with CSP. First, though, we continue this section with a description of how health professionals compared CSP with their previous practice. We note both common ground and variation in their understandings and their own contributions to enactments of CSP, as well as in their recognition of its advantages.

#### Commonality and variation in professional enactments of CSP conversations

Beyond ideas about how patients would ideally engage with CSP, there was much commonality in health professionals’ descriptions of how they conducted CSP conversations and how these compared with pre-PACE-D ‘usual’ consultations and annual reviews. Consistent features included CSP conversations: being allocated more time; being usefully supported by the visual (graphical) depictions of trends in patients’ biomedical markers that were shared in care planning letters (see Additional material); involving more listening to patients and less talking by health professionals; involving less issuing of ‘standard’ professional advice and more attention to patients’ daily lives; and (supposedly, but only occasionally) involving more leading by patients.

There were also, however, significant variations among professional accounts of their understandings and enactments of CSP. Some participants showed and developed a firmer grasp than others of values and communicative practices covered in CSP training, reflecting deeper commitment to the broad purposes and partnership ethos of CSP, following through more consistently on the recognition of patients as persons whose perspectives matter and the idea that the CSP conversation is a meeting between two experts, and describing more nuanced microskills of respectful, empathetic communication and collaboration. Table 1 summarises the accounts of four participants to give an indication of the range.

#### Benefits of CSP beyond improvements in health behaviours and biomedical markers

Between them, participants identified various benefits of CSP independent of (but perhaps intermediate to) changes in patients’ health-related behaviours and improvements in biomedical makers. Benefits identified by at least some health professionals included patients: being better informed; feeling less rushed, more listened to and better respected; sometimes appreciating that “the doctor is caring for them more, caring not for the disease but more emotionally” (HP Seow, w2); and sometimes being encouraged and enabled to feel and take more ownership of their conditions. Some health professionals also reported gaining a better understanding of each patient’s situation and perspective, which could in turn help them: (occasionally) solve previously baffling diagnostic puzzles; avoid unwarranted judgementalism; avoid alienating and demoralising patients; build rapport and trust; tailor suggestions more appropriately to patients and their situations; and “leave the door open” for people to come back for support in the future. Several doctors who stressed the value of these more emotional, relational and perhaps intermediate experiential benefits for patients also commented appreciatively on CSP as a better way of practising, an approach that reflected why they came into medicine, and one that could leave them feeling more satisfied with their encounters with patients and their work more generally (see Table 2).

**Table 2:** Broader benefits of CSP (beyond improvements to patients’ health-related behaviours and biomedical markers)

Illustrative Quotation	Summary
“It didn’t exactly go that well, but it was a case of when I looked at the patient’s problem in an open-ended manner and allowed her to speak, she said something like ‘this condition is something that I need to work on myself, it’s not something that other people can do for me’. And I think ‘Yeah’. And even though at the end of that conversation, there wasn’t a goal set or a plan made, I think it was a good step…. So previously it would be ‘OK, these are your results, these are your medications, I think we should shift, we should adjust the medications this way, I think these are what you should be working on’. Now we have flipped it around and ask ‘What is important to you?’, ‘What would you like to work on?’. I think that makes a lot of difference. When you throw the ball into the patient’s court, I think it forces them to think about what it is about their conditions that they want to work on. Whereas I find that if the doctor is the one telling you, ‘OK, this is what you should do’ the patient just reverts to being defensive, say ‘OK, I don’t want to do that’.” (HP Wu, w2)	When patients are asked openly, they may start to think about what is important to them and what they can do in relation to their condition (even if they don’t get as far as making specific plans).
“I will get to hear more about what really concerns the patients. Yeah. So maybe they feel that now they have more time, or more of an opportunity to voice out their concerns… or they were more likely to talk about it compared to a normal consult where they might feel they were a bit rushed… So I get to understand the patients more.… Spending a few minutes to find out what their preferences are, what their values are, this gives us a better idea of what we’re dealing with and then how to manage these patients subsequently… (HP Ho, w1) I do get a feeling that they feel that if for once they have been given the opportunity to talk more to the doctor, and let the doctor know about their side of the story, how they feel about things, they will get more satisfaction from that. Yeah. And the doctor is listening [laughs]. I think from the patient's point of view, they always like it when the doctor is listening [laughs].” (HP Ho, w1)	Patients have time and opportunity to voice concerns. Health professionals can better hear and understand. Patients may be more satisfied.
“What was interesting about this particular conversation was it turned to his things about [home situation], family matters. Things that weren’t purely about diabetes but they mattered to him. So it was positive in that sense because it allowed him to speak quite freely about these things… He is not with family, so the opportunity to be listened to would probably, I’m guessing, be quite precious to him… I listened to him, yes, I did, and I think he appreciated being listened to” (HP Ang, w1)	Patients can talk about what matters to them. Patients may appreciate being listened to.
“I have conducted some non-PACE-D consults where I was too engrossed in HbA1C… we have only that 5 minutes and I will just go straight to that point, ‘Okay, HbA1C is going up. It's not just going up, it's already in the terrible range’, and you could see how the patient responded and patient obviously had previous consults where the doctor has hunted them down, made them so demoralised and they just hate coming to the clinic… If we are going to venture into … asking them, ‘Why are you so upset?’ and things like that… that would need more time in the normal consults. So, often time we will be quite dismissive, … we will just say, ‘Okay, let's do this. Let's increase the medicine’ and things like that… Overall, it's yet another, I would say, negative consult, and patient get more and more depressed. So that doesn't help with the diabetes. But what I see from PACE-D … I don't see any patients going out of the room feeling downcast or adverse to this consult.” (HP Seow, w2)	Health professionals can avoid demoralising patients. Health professionals can investigate rather than dismiss sources of upset.
“I think the approach is sort of quite novel lah. Actually useful lah. Because I mean we used to think differently. So now at least we think that maybe we shouldn't be saying too much. Let the person talk. And if possible we try to sort of examine their ambivalence and if they got something, we don't argue, we try to roll with them. Maybe next time then there's more confidence and trust that - listen to them more, maybe it will help. So, CSP help us at least to learn some of these skills lah.” (HP Toh, w2)	Health professionals can avoid unfruitful arguing, work with patient to explore their reasoning, and build therapeutically useful trust.
“CSP makes me feel like I'm doing what a doctor should do... We have been trained to help people, we have been trained to listen to patients with open ended questions, to get a story out of them, to understand individual circumstances, to deliver individualized treatment… But because of systemic issues, because of timing issues, we have been always limited in the ability to deliver that. And therefore, our practice has evolved into a more paternalistic kind of approach, which, in my opinion is effective, is fast, but not the most ideal. CSP allows me to do what I've been trained to do: to work with people, not to dictate people's life.” (HP Boon, w2)	Health professionals can listen, understand and work *with* people in the ways they think they should.
“I have more time to show empathy to the patients and to really care for the patients rather than just mundanely go through the steps of the targets and the numbers. And often times, we can hear what patient is sharing. Even though it is not something to do with the illness itself, I feel that the consult is more joyful and it’s probably more education for me… I feel that if they benefit from it, even if just emotionally, I feel that that makes my day in that sense … I mean, you see patients 5 days a week, 8 hours a day. Sometimes, you can get so it's just like a chore. We just see patient and patient and patient - it's just like a factory. But this adds more meaning to what we do. And sometimes we just need to take it slow and value what we do… I think that helps with our own, I would say mental health, to a certain extent… maybe it helps us to look forward coming to work to spend time with patient… I mean it helps us to know that the patient knows that they are cared for… there is a proper doctor-patient relationship, there is rapport … So, that's way, way different from just seeing numbers, seeing patients as just clearing the queue.” (HP Seow, w2)	Health professionals can listen to and learn from patients. Health professionals can show empathy and develop rapport with patients. Health professionals can enjoy work more; this way of working can benefit professional wellbeing.

Variations in health professionals’ comments about these broader benefits of CSP seem to reflect variations in both their views of the purposes of CSP and their conduct of CSP conversations. It seems plausible at least that the broader benefits will be more readily realised and recognised when health professionals are more strongly committed, and have a deeper understanding of what it takes, to work in respectful partnerships, relate to patients as experts in their own lives, and take seriously patients’ own priorities for their health and wellbeing. The broader benefits will also carry more evaluative weight if these commitments are considered morally significant. We take the commitments to be consistent with the person-centred ambitions of CSP.

We will return to “Health professionals’ evaluations of CSP” below. For now, we consider the various challenges that health professionals experienced with CSP in practice.

### Perceived challenges to achievement of CSP’s potential

We have grouped the challenges that health professionals reported as relating to (a) patients and their contexts and (b) clinic systems and policy priorities. Quotations illustrating the various challenges and some different responses to them are presented in Table 3.

**Table 3:** Challenges to health professionals’ enactments of CSP and achievement of CSP’s potential

**Summary of challenge**	**Illustrative quotations**
***Variations in patients’ ability and willingness to engage***	
Many patients come to CSP conversations unprepared and perhaps reluctant to share their views	“So, some patients come in they might not be prepared, even though we mailed them the results, they [PACE-D coordinators] have told them that they are supposed to write out their thoughts to discuss with us, but many come in just [pause] is in blank lah. Yeah, so they are not prepared… they never really think of what are they going to do to help with their own conditions. Then I think that is a bit of the challenge as well. Even though we tried to stimulate them during the CSP itself, but some patient just doesn't really open up to me.” (HP Chen, w2) “If they really feel that they don’t want to know about how their conditions is, I will leave that and just continue back to the normal consult pattern, rather than continuing the CSP session, because I feel that it will be like hitting a stone wall for that session, lah. (HP Foo, w2) “Sad to say the majority of patients will come in, they have no idea what they are in for [despite the CSP process having been explained]. “Have you seen your results?” - “Yeah, I looked at my results.” “So what do you think about your results?” - “I don't know, you have to explain it to me”. And when you ask them about, “have you thought about your diabetes being poorly controlled…?” well, it's the same: the unpreparedness will show. And when you try to engage them into thinking about what they can do in their life, asking them to describe a day of their life, they sort of become hesitant, a bit withdrawn, like “This is not the kind of consult I'm expecting”.” (HP Boon, w2)
Health professionals or patients may lack the language skills needed for CSP conversations and important aspects of communication may be lost in translation when interpreters are required	“Most of the time we speak whatever language the patient prefers. That's the first thing lah. But that's where the problem is, because you know there's some things that is very hard to explain, and then sometimes when we try to transcribe, it actually loses the meaning, becomes sort of not what we intended for it to be… And you see for us, for example it's not only Chinese. We have to speak in Mandarin, Dialect, then Malay, ah? So I'm quite proficient … but it's that conversational, not at that kind of level where I can explain this word exactly, what it means”. (HP Toh, w2) “In a delivery model like that of CSP, the accuracy of message is very important … If I were to do a reflection and the translation comes out to be something else then it totally defeats that purpose. Yeah. So that is the biggest challenge I have when it comes to a language that's not native to me … I may not be able to deliver it effectively… The other way is also true. So when we ask patients, “What do you think about your diabetes control?” … when we talk to them in English, or if they're not efficient in the language that they use, they may just give us a very ambivalent answer. And that doesn't really reflect the concern or the worries that they have with regards to the condition because they don't know how else to express it.” (HP Boon, w2) “I can’t speak Malay, so if the patient can speak simple English, we can still communicate. But, if the patient cannot, I would need a translator. And a lot of times during translation, empathy gets lost I think. It’s very hard for me to tell the patient “I see you are really angry and then to get a translator to say that in Malay”. Yeah. So, I’m not sure what to do about that.” (HP Yeoh, w1) [*In response to a question about what could help ensure CSP works well for patients and health professionals*] “A translator that can translate empathy!” (HP Yeoh, w2)
Local medical culture has habituated patients to expect to be given medication and told what to do	“Some elderly… with a background of poorly controlled diabetes… not so forthcoming… of the mentality like “I’m here to get the medicine and being told what should be done” [and] when I try to ask in another way, then they kind of become a bit frustrated and they start to say, "Eh, you are the doctor, you tell me lah! Why am I here if you are not going to tell me?" (HP Lai, w2) “So the different thing [about CSP] will be like I ask them rather than I tell them lah… So I try to ask. “So you tell me, what you want to do?”, and “What are you going to do to achieve this or this?” So they will, they will say something, but … if at the beginning you ask, they are a bit scared. So you need to like a bit warm up. After warm-up, I find that's more better [laughter].” (HP Deng, w1)
***Organisational arrangements and policy priorities shaping delivery of CSP***	
CSP training scenarios were easier than those encountered in practice	“So, I thought that training was interesting, actually it was conducted very well. But it’s just that when I came out of the training and started practising it, it was really quite different.” [Interviewer: Can you say a little more about what’s the difference?] “The patients are not rehearsed [laughter]. The patients don’t come in with a script and then tell me what I want to know [laughter]. Right, it’s not like I ask a question and they will give me the answer that I want. So, sometimes they don’t want to talk or don’t answer my questions the way - they don't give me the answers that I was looking for. So then in those cases I will feel a bit stuck, so I’m not sure how to bring the conversation forward” (HP Yeoh, w1)
Clinic session pressures: long patient queues mean health professionals feel time pressures	“Because of the time constraints, lah, sometimes we have to rush through the consultation. So we can't really wait a lot and listen to all the patient's concerns … so sometimes we are rushing through. So some people, the proper CSP probably not enough time.” (HP Eng, w2) “If there is a lot of CSP patients for that day and there is a lot of patients waiting outside not for CSP, then I might actually shorten the CSP consult if I do not really have the time to go through. So yeah, so it might tend to go back to the old way of consult if let's say we are short of the time.” (HP Chen, w2) “More of a problem is time constraints. Because we have so many patients. So sometimes we tend to rush. … I must admit that sometimes, I tend to rush because I thought I still need to clear all the cases, the full pile of thing behind. Yeah. So that's one of the major challenges lah.” (HP Lai, w2) “While I try to honour the CSP process which is really listen to the patient, I think that there is an underlying part of me that tries to make it efficient.” (HP Ang, w2) “Sometimes I also get stressed about … whether I’m doing the CSP properly or whether I’ve been too rushed because there are ten patients outside and then I have to finish this CSP quickly… So sometimes I wonder whether if I had really more time, or less time pressure, whether the CSP would have gone a little bit better?… I think if given less pressure on the time and the queue, I would be in a better mood and then I can empathise better [laughs]. So empathy. And then I probably would have time to think about what they say… reflect better about what has been going on so far during the consultation. Yeah, otherwise sometimes I feel like there are a lot of things going on in my mind… during the CSP itself. (HP Yeoh, w2)
Appointment scheduling issues: CSP conversations are interspersed with usual consults (some health professionals struggle to change mindset or pace)	“Not every patient is PACE-D, so we have to change our mode of consultation in between. So the patients for PACE-D come, then we slow down. But then after patient go out, we have to go back to our usual way. Very tough.” (HP Chen, w1) “When we are seated in that hot seat, seeing that CSP patient, how prepared are we to really spend the time with them? … I think from my own personal experience there is a certain degree some sort of a barrier. Because it's about like switching head, as I am seeing my common queue patients, regular patients who – I might spend about five minutes with them and off they go. Suddenly a CSP comes in, I need to switch my head and say ‘This is a CSP patient, I need to sit down, calm myself down and not hurry, listen to them’. That's sometimes a bit difficult, especially when I am really in that mode of seeing patients.” (HP Boon, w2)
Teamlet working: staffing arrangements limit relational continuity, which can constrain conversation, impede development of rapport, and obscure health professionals’ view of how patients progress after a CSP	“It is not like a GP in the western countries where they have one doctor and every time they see the doctor it’s like they know them very well. For our patients here, we see doctors in a team and not every time is the same doctor. So they might not be too familiar with us to tell us such an in-depth history” (HP Foo, w2) “I’m sort of handicapped by the fact that I don’t always get to see the same patients because of the way that clinics are run. But I find that within the consultation there is a sense of positivity… I think that [challenging a patient’s expressed acceptance of poor diabetes control] is an option if you have a … certain rapport with the patients. In this case I think it was the first time I’m seeing this particular patient so I realised, it was probably not a good time of doing this. (HP Wu, w2) “Very rarely we see this kind of motivated patients. Unfortunately, I haven't got a chance to see back the patients who is so motivated, to see how is their HbA1c… probably it's followed up by my colleagues... So I don't know whether the HbA1c really improve after that, even though they do a full preparation.” (HP Chen, w2)
Medical record systems do not facilitate quick identification and review of notes from CSP conversations.	“We have no system to tag the patient to the doctor that saw the CSP. If we had, then maybe it's a bit better. Because sometimes, although the records are all there, it's very difficult to see through so many records when was the last CSP. Because in between, after the CSP, there may be a lot of other consultations for other things… I got to look at 10 different clinical entries before I reach the actual thing that I want for to launch our discussion.” (HP Toh, w2)
A systemic focus on biomedical markers and diabetes: Biomedical norms are prioritised in performance indicators and as the PACE-D primary outcome measure (a) this can be in tension with what matters to patients	”So, I would say that the sugar, pressure and the cholesterol is the 3 most important thing that I would try to like to make sure they are within the target first before I talk about others, other parameters. Yeah… because that's our KPI [laughs]. That's our clinic KPI.” (HP Chen, w2) “We want to succeed. And if you see ten patients and everybody ends up with a HbA1c of 8 and your friend ends with a HbA1c of 7, definitely you will think something is wrong.. That means you won't be CSP-orientated, but more medications driven lah… Of course, the actual clinical parameters is very important lah. But at the end of it, I think if the patient goes off satisfied, meaning that he thinks that you listened to him, you tried to do the best for him, I think that is success lah. Of course, it's not measurable, but there is a form of success to me.” (HP Toh, w2) “One of the questions [doctors were] concerned about when it comes to leaving things to the patients is that while we have our clinical performance indicators, quality indicators to look after, if we don’t push, and patients don’t meet this, then the scores all suffer and that kind of thing. So that was one of the concerns … I realised … that if you’re trying to push the patient to go a mile, okay and the patient wouldn’t go along with it, then the patient is at zero. But if you make this a more a collaborative thing and the patient says ‘Oh, okay I may not be able to go a mile with you, but I’m willing to go half a mile’ then the patient is half a mile further from zero.” (HP Wu, w2) “[The results letter] sort of spells out for the patients what their goals would be… because… green is the goal that each table is pointing the reader to. And in fact all the goals in the traffic light are very biomedical, like weight target, blood pressure target and HbA1c target. So very naturally, when a person goes to the page to talk about what your goals are, they will tend to think about medical targets for their goals. And that's why, when I ask them about a life target for their goals, they seem as if it's a long shot away from what they were expecting any doctor to ask them… So I think that frames how our patients think about goals… Whereas when I speak to them, I'm trying to get them to think about the bigger story of what they really want and how these are really just surrogate measures of how to get there. So it does constrain.” (HP Ang), w2
A systemic focus on biomedical markers and diabetes: Biomedical norms are prioritised in performance indicators and as the PACE-D primary outcome measure (b) this can raise questions of what is relevant to include in a CSP conversation.	“[Hearing long stories] is something that I struggle with I would say. Because one of the things in polyclinic is that speed is a thing. And I think it’s also something in the training of a physician… being able to come to a diagnosis and offer a solution quickly is considered a good thing, right? … So, I think this is something I struggle with, when the patient starts the story and I sort of think I already know how the story ends and all that … So I suppose I learn not to cut them off, just listen and yeah, see where it takes you” (HP Wu, w2) “So the time that has been budgeted ends up being about talking about life, for example… Is it a loss? No, I don’t think so. I think it’s just about getting to know my patient better. And, yeah, we don’t exist for diabetes alone, I suppose, as doctors” (HP Ang, w1) “I basically base my questioning, perhaps not as widely as I expected it to be in terms of - for example, when it comes to the goals of living better with diabetes, which is the language we use, I kind of narrow it down because at the end of the day, it turns out to be that the reasons why people want to be healthy turns out to be - in [my] mind … about three, four or five reasons … (avoidance of complications… avoidance of being a burden to the family… independence and freedom and being able to do what I want to do… seeing the grandson grow up – the family relationship matters…). So when … I want to ask for the goals, if the patient is a bit not in the habit of reflecting or thinking of possible answers, I would, after a bit of waiting, give some examples… I found that if I talk about these life goals as opposed to disease focused goals, they tend to find some kind of synchronicity … For the blue collar workers, the elderly types, sometimes they are not given to thinking about these things… and maybe the language to describe their goals may not be as rich… I try to solve the problem with them by giving them some model answers I know of: “Do any of these apply to you?” (HP Ang, w2)
A systemic focus on biomedical markers and diabetes: Biomedical norms are prioritised in performance indicators and as the PACE-D primary outcome measure (c) this can raise questions about where it is acceptable to end a conversation.	*This example involved a woman who attributed a significant worsening of her HbA1c to the fact she had been making a herbal drink for her family because of the haze (high levels of air pollution) and had to add sugar before they would drink it. She realised while telling the story that she could take her portion out before adding sugar, but seemed disinclined to make a change:* “So at the end of it, she said “I’m happy with my blood sugar control.” Even though I wasn’t. And “I’m happy with my lifestyle.” … I suppose pre PACE-D it would have been a bit harder for me to accept… I mean there’s like “You shouldn’t be happy with this”, right? I could understand if you’re happy with your lifestyle, but you shouldn’t be happy with your results. I think post PACE-D there is - it changes I suppose the clinician also. So, I find it easier to accept because I sort of understand that there is no point in trying to force the patient to change his or her mind. Because it probably wouldn’t work. And … I think … from what she said and probably by the way she said it, I also understood that … maybe she’s happy with the lifestyle, but I think she probably wouldn’t be happy with her control. It was probably something she said in the sort of self-rationalisation, sort of denial thing. But … with the PACE-D training, I thought it wouldn’t be (how you say?) profitable to push the point, you know. It was just enough to accept that, and to not close the doors and keep the conversation going. So, I think that’s something I learnt from the PACE-D year of care training” (HP Wu, w2)

#### Variations in patients’ ability and willingness to engage

Participants reported that many patients did not open or read, let alone reflect and write in care planning letters before their appointments. In the early stages of PACE-D they estimated that up to 30% of patients did not read the letter at all. They reported that this improved after it was agreed that PACE-D coordinators would check and provide more active assistance when patients came into the clinic (see below) and as patients became more familiar with the process.

Health professionals further experienced many patients as reticent or lacking confidence to engage in conversation about their diabetes in the context of their life. They mostly explained this with reference to some combination of language diversity, education and culture, although some somewhat judgementally also suggested a lack of interest on patients’ part, and some more sympathetically recognised that a long social history of medical practice *not* showing interest in patients’ perspectives was partly to blame.

Care planning letters were issued in whichever of three languages (English, Chinese or Malay) a patient preferred. However, many older adults in Singapore received little education and literacy rates among people over 55 are relatively low [25]. Health professionals reported that some patients struggled to understand the information provided - despite the visual depiction of trends and traffic light colour coding. A limited understanding of body and health concepts could reduce people’s scope to reflect on their test results and generate ideas for improving them. And although the additional time allocated to CSP conversations allowed health professionals to explain and discuss the information provided, CSP training had encouraged the view that the advance sharing of results should mean less consultation time was spent on this and more on hearing patients’ perspectives and supporting them to set goals and develop action plans.

All participants were used to conducting consultations in at least two languages, but several explained that language skills that sufficed for ‘usual’ consultations were not necessarily up to the more nuanced demands of open, broad-ranging and potentially emotional CSP conversations. Patients, too, might have limited ability and confidence to express complex thoughts and feelings in the language of the conversation, and the limitations of interpreters could be particularly problematic for CSP consultations.

Several participants commented that aspects of Singaporean or more broadly Asian cultures meant many people were not used to being asked for their opinion, particularly in healthcare contexts where (to a greater extent than in the UK or more broadly Western cultures within which CSP originated) doctors are deferred to as authority figures and families and family hierarchies are influential in healthcare decision-making. The CSP approach represented a big cultural-behavioural shift within polyclinic service provision. People with diabetes who had attended clinics for many years were likely to be strongly habituated to ‘usual’ consultation practices in which they were assessed quickly and sent away with medication and instructions. While all participants showed some awareness of the diversity of patients’ educational and cultural backgrounds and socioeconomic circumstances, some reflected these less clearly and sensitively than others in their accounts of CSP conversations. Not all recognised that patients might need substantial encouragement and experience of a few rounds of CSP to adapt to the expectation that they engage as ‘equal’ partners with the health professional in conversation. Not all seemed particularly able or willing to cede their biomedical priorities and authority to facilitate such partnerships.

The reticence and perhaps limited ability of many patients to engage in CSP recurred in participants’ accounts as a challenge that would tend to limit achievement of the approach’s theoretical promise, particularly in relation to biomedical health outcomes. Some participants quite quickly got “stuck” when patients volunteered little in the way of ideas and opinions (this was perhaps more likely if the health professionals were encouraging a focus on biomedical test results). These health professionals described often ending up with a CSP conversation that was more like a “usual consult”. Those who were also less inclined to recognise other potential benefits considered patients who were less able or willing to engage “less suitable” for CSP. Other health professionals, in contrast, were apparently more determined to hear and work with patients’ perspectives and persisted as constructively as they could with whatever little they could elicit from patients’ initial responses, expressing hope that patients would become more confident and open with more experience of the CSP approach. A few described developing and refining their questioning over time to help patients reflect on and express what was important to them.

#### Organisational arrangements and policy priorities shaping delivery of CSP

##### CSP training, professional huddles and PACE-D coordinator support

Health professionals spoke positively about the CSP training they received and the promise of the approach that it conveyed. Some described starting to use some of the ideas and techniques they learned immediately – before patients had been recruited for PACE-D. There were, however, some challenges associated with the training and support provided for health professionals which we note here.

For some participants, a time lag between the training and their first formal CSPs with patients meant they perhaps forgot some ideas and techniques and lacked confidence before they started in earnest. Several participants also reported “getting rusty” during periods of leave or secondment and having to rack their brains to remember what CSP entailed when they returned to the teamlet.

Some health professionals commented in interviews that the scenarios used in training were simpler than those encountered in the clinic where patients did not all follow an ideal kind of script. During the final huddle at which these findings were shared, members of the training team shared a reflection that perhaps they had painted too rosy a picture and so set up too high expectations during the first CSP training session.

The huddles, intended as supportive sharing and learning opportunities, were scheduled for lunchtimes, with food provided. Health professionals typically arrived late as morning clinic sessions ran over time, and they were often visibly tired. In the first huddles, a few practical challenges and questions were raised and addressed, including how to encourage patients to engage with their results information ahead of the consultation (which led to an arrangement that PACE-D coordinators could print a copy of the letter when patients checked in for their appointment and support them to review test results and think about questions or goals before seeing the doctor or nurse). In later huddles, partly in response to insights emerging from this study, CSP trainers shared short videos and presentations encouraging reflection on empathy and goal setting (including attention to goals beyond the biomedical) and examples of completed goal and action plan pages.

##### Appointment scheduling and clinic session pressures

Both polyclinics scheduled all CSP conversations for two specific weekdays, but routine consultations, including walk-in appointments, were also allocated to these clinic sessions. Some participants found the intermittency of CSP conversations within clinic sessions challenging because they needed to keep switching gears for the change from “usual” consultations.

The workload pressure generated by long patient queues was a strong theme in all interviews. In principle, more time was allocated for CSP conversations (fewer appointments were allocated to clinic sessions with CSPs). In practice, however, participants were often uncomfortably aware that their teamlet partner would be struggling with a long patient queue while they took 20-30 minutes for a CSP conversation – and they would also return to face the queue. All participants admitted at least minor shortfalls from what they understood to be good enactments of a CSP conversation when feeling this pressure. This was well summed up by HP Boon’s reflection that “We may short-change our CSP patients to a certain extent” (w1), but the shortfalls apparently varied in the extent to which they undermined the values and ethos of CSP. When keeping an eye on the patient queue resulted in sometimes rushing a bit, being less fully empathetic, or not working through the steps of goal setting and action planning with patients with well-controlled diabetes and no significant concerns, the shortfalls were arguably less significant than more routinely “cutting short” what patients were saying and “reverting back” to usual consults along the lines of telling patients what to do and being summarily dismissive of those not ready to act on their diabetes as health professionals would prefer.

##### Teamlet working and record systems: challenges to continuity

Patients’ CSP conversation appointments were to a teamlet clinic session, not to a health professional. Teamlets were notionally staffed by two doctors and a nurse, but various leave and secondment arrangements (especially through COVID-19 disruptions) meant multiple health professionals rotated through the teamlets providing CSP. PACE-D leaders had trained sufficient health professionals in CSP to cover staff movements, but staffing changes limited continuity of professional-patient pairings before and after CSP conversations.

Some health professionals thought the lack of relational continuity could contribute to patients’ reluctance to “open up” in consultations. It could also limit scope to build the kind of rapport that some participants considered necessary to enable them to challenge patients in ways consistent with the empathetic and collaborative ethos of CSP.

Challenges to continuity were exacerbated by limitations of the medical record-keeping system (including after the introduction of the new system). Although the records of patients enrolled in PACE-D were flagged, some participants found it hard to identify which of a patient’s previous appointments had been for a CSP conversation, so struggled to find notes about important information patients might have shared and goals and action plans agreed. Several participants admitted that appointments after the CSP conversation were not always recognised as follow-ups. This could limit continuity of approach as well as continuity of information: a few participants expressed concern that an ethos of empathic engagement with patients’ perspectives was not always sustained as not all colleagues were inclined to extend their use of open questions, affirmation and reflection to consultations other than CSP conversations.

In addition to potentially diminishing patients’ experiences and the effectiveness of CSP, limited relational and informational continuity also obscured health professionals’ view of how patients progressed, potentially depriving them of positive motivational reinforcement when their efforts with CSP were bearing fruit.

##### A systemic focus on biomedical markers and diabetes

Although CSP training emphasised working with patients as persons and stressed the value of health professionals attending to patients’ concerns and priorities relating to their diabetes and broader wellbeing, participants were aware that the primary outcome of the PACE-D trial was HbA1c, and that their teamlet and polyclinic performance was judged on HbA1c and other biomedical markers, with implications for salary bonuses. Some participants also recognised that several features of the care planning letter (which they often referred to as the “results letter”) tended to encourage patients to concentrate on biomedical markers.

Health professionals managed these potentially competing demands with different emphases. This had implications for their view of what was important for them to hear in CSP conversations as well as their assessment of what was agreed with patients and of subsequent outcomes.

Health professionals who conveyed a narrower view of the purpose of CSP worried that inviting patients to talk about their concerns could expose them to a long list of issues to be addressed, many of which would be irrelevant to the patient’s diabetes or were not things health professionals could help with. These health professionals sometimes acknowledged that it could be useful to find out about a person’s daily routine and key concerns to be able to tailor their professional advice about diet and exercise (to help ensure action plans to achieve biomedical goals were feasible), but their reasoning about this was somewhat narrowly instrumental.

In contrast, health professionals who put more emphasis on the idea that the CSP conversation should focus on the person and what mattered to them seemed also to value learning more about patients’ lives and perspectives. They appreciated getting to know people better and being able to empathise and allow a patient to feel “more human” in the conversation. Some reported a range of benefits of this broader understanding contingent on the situation. We noted above that some participants had developed strategies to “fish out” a sense of patients’ priorities even from those who seemed less able or willing to respond to unfamiliar requests to communicate their goals. A few of those with broader views of the purpose of CSP had explicitly formulated questions to encourage patients to think broadly about what mattered in their lives.

Another key point of variation was whether and to what extent health professionals saw a need for a clear action or action plan to conclude a CSP conversation – particularly to know or have evidence that they had done something to address poor diabetic control. Some participants seemed determined to have a professional or biomedical last word if a person appeared disinclined to do anything for themselves to improve on poor diabetes control, reverting to “usual” consultation practice with an emphasis on their own perspective and reiteration of their advice that the patient really should be taking action. For others, in contrast, CSP training had taught them or supported a previous view that it was not necessarily a professional failure to do nothing, or not emphasise a point that a patient already knew; in some circumstances the kinds of outcomes identified in Table 2 were all that could realistically be achieved at the time.

### Health professionals’ evaluations of CSP

We have seen that health professionals varied in terms of how they interpreted and enacted CSP, including in response to the challenges posed in the polyclinic context. In this section, we examine their broader evaluative judgements.

When asked for their overall evaluations of CSP and thoughts about whether CSP should be extended beyond the PACE-D study or to other polyclinics, health professionals were generally cautious in their responses, expressing uncertainty and giving qualified “it depends” type responses. There were various reasons for this, not least that they all awaited the results of the PACE-D trial for evidence of any population-level effect of CSP on HbA1c. As noted, participants only saw clear improvements in biomedical markers in a minority of patients, and limited continuity of care obscured their view of how things went for many of the patients with whom they had CSP conversations. Their interpretation of the lack of evident quick biomedical wins varied, and although they often recognised some other benefits of CSP relative to “usual” consultations, they attached different, sometimes uncertain, levels of significance to these.

No participants expressed any concerns about patients being worse off for being offered CSP (except when considering what would happen if they persistently harangued reluctant patients for ideas and goals – which none of them reported doing). A few, like HP Wu, expressly noted that “there’s never been a case where it is worse than the normal consult” (w2).

For some health professionals, however, the benefits of CSP, whether narrowly or more broadly construed, came at the price of significant professional effort. It could be difficult to learn and remember how to conduct CSP conversations, some struggled to switch between CSP conversations and usual consultations, and the pressure of the patient queue added stress to a longer CSP conversation. Some health professionals became rather demoralised when they did not see many patients come as “prepared” for as hoped for CSP, when they kept getting “stuck” when patients’ engagement was limited, and when fewer patients than they had anticipated showed clear behavioural or health status improvement at follow-up.

Significant administrative and PACE-D coordinator resources were invested in producing and sharing care planning letters with patients and supporting CSP processes. CSP conversations also had opportunity costs in terms of the professional consultation time available for other patients. Resource considerations featured in several “I’m a fan of CSP, but” and “it depends” evaluative statements.

In addition to the proportionality of benefit and effort or cost, overall evaluations of CSP also depended on what health professionals used as their reference comparator. When compared with rare but striking best examples of patient behaviour change and biomedical improvement, health professionals’ more routine experiences with CSP could seem unsuccessful, especially if success was measured over a short time horizon from one CSP conversation. When compared with usual consults, however, they could seem significantly better – especially when richer enactments and a broader set of potential benefits were considered and a longer timeframe was allowed for the realisation of potential health benefit.

Opinions about whether CSP should be extended beyond the PACE-D trial could also depend significantly on which patient groups were considered (or proposed to be included in the future), and how situational challenges and variations in professional practice would be addressed. Some health professionals advocated offering CSP to a more limited group of patients, and perhaps concentrating its delivery among a smaller group of health professionals.

## Discussion

The health professionals who participated in the PACE-D trial have contributed to the implementation of CSP in a busy public sector primary care environment, and they sustained it through COVID-19. This is a significant achievement, especially given the documented pressures of working in Singapore’s polyclinics [15, 26]. All health professionals experienced practical challenges associated with local patient demographics (including a significant inter-generational education gap) and features of healthcare systems and culture. They responded to these, and more generally enacted CSP conversations, with varying levels of adeptness and fidelity to the person-centred commitments of the CSP approach. Although all saw positives to CSP, the range of perceived benefits varied, and some were hesitant about the circumstances under which it would be appropriate to extend its use beyond the trial.

The experiences and insights that these health professionals shared allow us to extend theoretical understanding of the significance of health professionals’ interpretive emphases of CSP and the complex interactions of these with external contextual challenges. Our analysis of this, which we report below after a brief discussion of the strengths and limitations of our study methods, is particularly relevant for the development and evaluation of future efforts to introduce CSP and other forms of person-centred support for people with long-term conditions, especially in contexts where prevailing healthcare and broader social cultures include a strong deference on (bio)medical authority. Along with the more evident scope identified in the Results section to modify local systems to address some of the challenges participants in this study reported, this analysis informs the recommendations outlined in Table 5.

### Strengths and limitations of methods

In the context of the PACE-D trial, we observed CSP training, interviewed all doctors and nurses who conducted more than a handful of CSPs (half of them twice) and observed a series of professional huddles. We generated a rich dataset that supported detailed examination of both commonality and variation in health professionals’ interpretations, enactments, experiences and evaluations of CSP, and enabled us to consider how these developed (or not) over time as familiarity with the intervention grew. What we heard from the health professionals involved in CSP conversations was consistent with what we learned from PACE-D coordinators and resonated for the CSP trainers (who also led the huddles). Our findings and key analytic points were endorsed by participants when we presented them at a PACE-D huddle.

Important limitations were that we did not observe clinical enactments of CSP and did not attempt to link our interviews with health professionals to interviews with patients. Because the interviews were conducted in the context of the ongoing PACE-D trial, experiences of trial participation inevitably overlay participants’ experiences of CSP in clinical practice. This might have increased some participants’ emphasis on biomedical markers and experiences of tension between biomedical and patient priorities. It does not, we believe, undermine the robustness of our analysis and recommendations.

### Contribution to theoretical understanding

As briefly noted in Background and Methods sections, our attention to health professionals’ interpretive emphases and the implications of these for the success of CSP was informed by previous literature highlighting: variations in how health professionals interpret and enact ideas relating to patient empowerment and enablement [18, 19]; the significance of narrower and broader views of purpose in healthcare support for people with long-term conditions [4, 19, 20]; and the tensions that health professionals must somehow navigate when they try to work responsively with what matters to patients in the context of systems which prioritise biomedical outcomes and foster a professional reluctance to endorse behaviours that do not help with health conditions [21, 22].

Our study supports and enables us to extend this previous work with more attention to temporal and teamworking considerations and the operation of interpretive emphases within complex and value-laden systems of healthcare provision. We develop here an analysis of how the interpretive emphases, value commitments and skill repertoires that health professionals bring to CSP interact with practical-situational challenges external to the health professionals involved. We also outline the implications not only for how health professionals conduct CSP conversations and follow-up consultations, but also for their scope to develop their skills with practice, for their experiences of CSP and motivation to use it over time, for the overall impact of CSP, and for health professionals’ evaluations of the approach.

Table 4 identifies variants of some key features of health professionals’ interpretations, value commitments and skill repertoires that are less and more conducive to broadly effective enactments and positive evaluations of CSP among diverse patients. The two columns are not intended to characterise the full spectrum of possibilities within each row, and we are not saying that each health professional can be simply and confidently categorised in just one of the two columns. However, the features listed in each column were all illustrated in our interview data and tended to cluster within health professionals’ accounts (see also Table 1). We outline their implications here.

**Table 4:** Interpretations and skill repertoires that impact enactments and evaluations of CSP

	**Less conducive to broadly effective enactments and positive experiences and evaluations of CSP**	**More conducive to broadly effective enactments and positive experiences and evaluations of CSP**
View of purpose of CSP	Improve HbA1c and other biomedical markers or risk factors for diabetes, supporting patient to work on these.	Improve health and broader wellbeing, working with person’s own perspective on these. (This can include, but is not limited to, biomedical markers and risk factors).
View of CSP conversation as ‘causal’; expected timeframe of success	As a discrete intervention, with an expectation that it will impact directly and relatively quickly on relevant behaviours and (biomedical) health outcomes.	As an intervention that is part of a long-term process in a complex social environment. It may have various benefits, but impacts on health may take a long time to become evident.
Understanding of how motivation features in CSP	Patient should come in prepared and motivated to improve their health; practitioner works with a motivated patient to set goals and action plans to improve their diabetes.	Patient ideally comes in prepared and motivated to improve their health, but practitioner role includes working to identify and develop patient’s motivation and support them, when appropriate, to set goals and action plans to improve some aspect of their health and wellbeing that matters to them.
Why listen to patients and find out about their lives?	Attend to patient’s views about their test results and elicit practical details of their life in order to identify scope for them to act to improve their diabetes.	Attend to patient’s views about their diabetes, perspectives on what matters and key features of their life in order to understand them better and to find appropriate ways to support them to address their own priorities for diabetes or broader health and wellbeing.
Emphases in professional interest	Strong interest in disease, bioscience and/or epidemiology; less drawn to psychosocial aspects of healthcare	Strong interest in people and the psychosocial aspects of healthcare as vital for good use of biomedicine
Relevant prior training	No or little prior training with motivational interviewing or similar.	Some (perhaps extensive) prior training and experience in motivational interviewing or similar.
How CSP relates to previous practice	CSP perhaps involves a significant change, but the difference is seen mainly in terms of allocated consultation time, consultation stages, the kind of questions the health professional asks and the balance of who talks.	CSP allows space for and perhaps gives a formal structure to approaches to working with patients that were already of interest or being tried. Or CSP involves a significant change in approach involving a shift in values towards more sharing and a greater appreciation of working with what matters to the patient.
Presumptive comparator in evaluative judgements of CSP generally	‘Ideal’ account of CSP Or The most positive examples of substantial improvements in patients’ biomedical markers following CSP.	Pre-CSP arrangements, including ‘usual’ consultations.

We take the ‘less conducive’ side first. Health professionals who view improvements in the biomedical markers of diabetes as the ultimate purpose of CSP, and who expect CSP as an intervention to deliver those improvements as a result of patients conforming to expectations that they will actively prepare for and engage in consultations and make and follow through on plans to improve their health, will have relatively little scope to see their efforts with CSP as successful when CSP ‘works’ like this for only a minority of patients. If they further (perhaps relatedly) think that CSP will only benefit patients who come to a CSP conversation already motivated to act to improve their diabetes, health professionals can quickly become pessimistic about its prospects when relatively few people arrive having written in their care planning letter or otherwise willing, ready and able to contribute ideas about what they can do. The value of CSP for people whose diabetes is currently well controlled may also seem questionable.

If health professionals also consider what is unique or importantly different about CSP primarily in terms of process steps, they are less able to reason confidently about when it is appropriate to ‘skip’ certain steps – for example if goal setting and action planning is not a top priority given a patient’s current circumstances and concerns (a tick box process orthodoxy can work against the flexible responsiveness that CSP requires).

If health professionals think the main reason for listening to patients is to identify scope to tailor their advice and better persuade patients to change their behaviour to improve their diabetes, then other things that patients tell them, including about other conditions, financial worries or other social concerns, can seem irrelevant to their conversations. If health professionals feel obliged to address these concerns once raised, however, more open questioning risks adding to their workload or sense of failure. The investment of professional time and energy may not seem worthwhile, especially in busy clinics and amidst concerns about the opportunity costs of the approach.

If health professionals have little prior interest or learning to support their attention to patients’ psychosocial issues, they can get “stuck” quite quickly when patients are not very forthcoming in conversation. If they lack the foundation to develop their communication skills and approaches beyond those covered in the CSP training session, this can contribute to a downward spiral of professional enthusiasm and more negative evaluations of CSP.

In contrast, on the ‘more conducive’ side, health professionals have much more scope to see success if they have a broader and more flexible view of the purpose of CSP, including the idea that the focus of the CSP conversation and any resulting actions should be set or strongly influenced by the patient and what is important to them. Even if a particular CSP conversation cannot be considered an unequivocal success, a broad view of purpose and valuing of the emotional, relational and perhaps intermediate experiential gains from CSP can help health professionals to accept (rather than view as a failure) when CSPs result in little immediate action or evident progress on a professionally concerning health issue. If the patient’s concerns and considered priorities had been heard, the patient was aware of any serious professional concerns, and the door had been left open for further discussion in the future, agreeing to keep important issues in view might perhaps be as far as they fruitfully could go in that consultation.

If health professionals have more realistic expectations about the time frame over which any general, population-level health benefits of CSP are likely to emerge and if they recognise that these depend on multiple other influences beyond the CSP conversation (including follow-up consultations as well as patients’ circumstances and behaviours, and the not entirely linear causal connections between behaviours and health indicators) they are also less likely to be disappointed about what CSP achieves.

The scope to see some success even when patients do not engage with CSP as fully as hoped or do not achieve as much health improvement as the ‘best’ examples is more likely to keep health professionals enthusiastic and motivated to continue with the approach. A sense of potential to learn and improve on enactments of CSP can also help in that regard. In our study, health professionals who expressed strong interests in the psychosocial aspects of primary care or in “people more than disease”, and those with more advanced communication skills and familiarity with reflexive, practice-based learning were also more inclined and confident to develop and refine their enactments of CSP, for example by trying out different questioning strategies. A combination of a broad sense of purpose, commitment to be flexibly and respectfully responsive to patients, and good initial skill repertoire facilitated positive development of practice over time.

Our study demonstrates the complex interactions between health professionals’ interpretive emphases, value commitments and skill levels and the various challenges associated with diverse patient situations and organisational systems. It identifies potential for negative feedback loops to develop from narrow interpretations of purpose and limited initial skill sets, and for positive feedback loops to develop from broader interpretations of purpose, a stronger initial skill repertoire, and confidence and support for reflective learning and skill development.

In healthcare contexts in which there is limited relational continuity in care, significant variability between the various health professionals involved in a team providing CSP will reduce the chance of any one patient from benefitting richly from a CSP conversation and from effective follow through from that conversation. Limited relational and informational continuity also reduce the scope for health professionals to see benefit from their efforts with CSP and make it less likely that patients become more familiar and comfortable with CSP approach over time. Divergence from key CSP principles is thus likely to be particularly detrimental to the overall success of the approach in such circumstances.

### Implications for policy, practice and research

There is clearly scope to address some of the challenges for CSP associated with organisational arrangements. Our analysis also indicates that implementation of CSP could be strengthened by constructive attention (and beyond initial CSP training) to those aspects of health professionals’ interpretive emphases that are less conducive to broadly effective enactments of CSP, and perhaps by ongoing support to foster the further development of communicative microskills. Constraints associated with patients’ educational levels and cultural backgrounds are less directly modifiable within health service provision. However, work on healthcare systems and professional skills and attitudes to help ensure patients are more consistently supported with flexible encouragement to ask questions, express their views and concerns, and experience positive responses to these might be beneficial for the longer as well as shorter term.

We have developed recommendations for organisational systems and policy development, for CSP leads and trainers, and for research. These are presented in Table 5, where we also note that informal sharing of findings from this study has already led to several recommendations being implemented in Singapore.

**Table 5:** Recommendations developed from this study

**Recommendations for organisational systems and policy development:**
• Investigate scope to improve language matches between health professional and patient for CSP conversation appointments • Review appointment systems to improve relational continuity through CSP consultations and follow ups • Consider establishing dedicated clinics for CSP conversations or clearly demarcating and protecting a block of time for these within mixed clinic sessions. • Review the medical record system for scope to facilitate identification of notes about CSP conversations and follow-up (progress review) discussions • Review Key Performance Indicators and associated incentives to reduce emphasis on biomedical markers and reflect commitment to person-centredness and broader wellbeing^a^ • Strengthen post-training support for health professionals delivering CSP (see below) • Perhaps consider staff assignment to CSPs to reflect interests and skills^b^
**Recommendations for CSP leads and trainers**
• Review the CSP letter as modified for Singapore with a view to more clearly encourage patients and health professionals to reflect on what matters in the patient’s life and for their health and wellbeing (including psychosocial issues beyond the biomedical markers for which test result trends are provided) • Refer to the CSP letter as a ‘preparation’ or ‘planning’ letter or similar, rather than as a ‘results’ letter to help encourage preparation and with a broader focus.^b^ • In training and follow up support for health professionals who deliver CSP: ◦ Prepare health professionals more explicitly and practically for some patients coming to CSP conversations ‘unprepared’ and, for various reasons, being not very forthcoming with their ideas^b^ ◦ Encourage health professionals to check and reflect on their interpretive emphases and if necessary consider whether a shift to positions more conducive to broadly successful enactments of CSP would be appropriate. Table 4 could be the basis for a tool to support this. Meanwhile we note it might be particularly important to: • Debunk expectations that CSP will mostly go according to the ‘ideal’ model with quick wins in biomedical improvements^b^ • Encourage health professionals to keep in sight a bigger picture of how diabetes impacts patients, to adopt a broad view of the purpose of CSP (enabling people to live well with their condition) • Encourage recognition and appreciation of the ‘softer’ relational and experiential benefits of CSP – both in their own right and as possibly intermediate to longer term health benefits. • Promote the underlying ethos of CSP as valuable in its own right and attend to this as the basis for the ‘usual’ process steps (a hollow or inflexibly dogmatic tick box approach to CSP steps may be counterproductive, there needs to be an underpinning interest in the person’s wellbeing and life and orientation to a collaborative and continuing supportive approach) • Discourage viewing the CSP conversation as a ‘one-off’ intervention • Encourage recognition that one can do ‘a good job in the circumstances’ • If possible, offer occasional ‘peer review’ by a skilled trainer who can observe consultations and support individual health professionals to reflect on and improve their practice
**Recommendations for interpretations of trial findings and further research**
• Be aware of outcomes (including experiences that may mediate longer term health outcomes and are broadly relevant for wellbeing) that are not assessed • Be aware of varying fidelity to the intervention (and recognise that the adverse effects of some shortfalls in fidelity may be compounded in some circumstances) • Be aware of potentially modifiable systemic challenges and shortfalls in some professional enactments of CSP conversations and follow up that have likely limited the impact of CSP on health outcomes. • When inviting or interpreting health professionals’ evaluative comments and thoughts about whether an approach they have tried should be extended, check their reference comparator (an unrealistic ideal or previous usual practice?). If possible elicit and bear in mind how they have understood and enacted the approach, and in what circumstances. • Be aware that simple rating questionnaires about the value of CSP are potentially misleading if health professionals are making different assumptions about patient populations, working contexts (including organisational support) and the skills of the health professionals involved. • Qualitative studies of health professionals’ perspectives can add value

^a^This is now being reviewed in Singapore

^b^The trainers involved in this study now more explicitly prepare health professionals for the fact that not all patients will reflect in advance of the CSP conversation or respond expansively to their questions. They are also modifying the language they use to emphasise insights generated from this study

## Conclusions

CSP has been implemented and sustained in two busy polyclinics in Singapore. Health professionals’ accounts have illuminated some important challenges and variability of enactments and experiences of CSP. They supported an analysis that highlights the practical significance of health professionals’ interpretations of the purpose of CSP and their valuation of relational, emotional and other perhaps intermediate experiential outcomes as well as the more typically considered behavioural and biomedical outcomes. Health professionals’ interpretations of CSP, along with their communication skill repertoires and support for reflective experiential learning, interact in complex ways with other features of healthcare systems and diverse patient-circumstance scenarios. They warrant careful attention in efforts to implement and evaluate person-centred support for people with long-term conditions.

### Supplementary Information

**Additional file 1: Table 1.** Examples health professionals offered of CSP consultations that went well.

**Additional file 2.** Sample Care Planning Letter (blank), English.

## Data Availability

The datasets generated and analysed during the current study are not publicly available due to the agreement made with participants to protect individual professional privacy. They may be available from the corresponding author on reasonable request subject to approval from the Yong Loo Lin School of Medicine, National University of Singapore.

## Abbreviations

CSP: Care and Support Planning
HbA1c: Glycosylated haemoglobin
HP: Health professional
PACE-D: Patient Activation through Community Empowerment/Engagement for Diabetes Management
