# Supplementary material for: Implementing and evaluating care and support planning: a qualitative study of health professionals’ experiences in public polyclinics in Singapore
Source: BMC Prim Care. 2023 Oct 19;24:212. doi: 10.1186/s12875-023-02168-5 (PMC10585850; doi:10.1186/s12875-023-02168-5)
Supplement: Supplementary file 1 — Additional file 1: Table 1. Examples health professionals offered of CSP consultations that went well. [file 12875_2023_2168_MOESM1_ESM.docx]

Additional Tables

Additional Table 1: Examples health professionals offered of CSP consultations that went well

| Health Professional (pseudonym) | Account offered as examples of CSP consultations that went well | Outcome |
| --- | --- | --- |
| HP Khay | “Generally, I find that CSP goes really well if the patient is prepared … they have written their goals and everything. So perhaps one encounter I can remember … the patient … came in pretty stable to begin with … he was very motivated to come off the diabetic medication. So he want to improve even more so that he could actually convince us to take him off the medication. So, he had goals that he had set… and had some concrete plans. Yeah. Then it was easy to talk with him and it went really well. There was no resistance … didn't take too long as well … because he already had thought through most of the things that he wanted to do.” (w2) | Reduced need for medication |
| HP Deng | “This morning I have seen someone. … His plan was lose about 10kg, but that time when he jot down the target, he did not say how long. So, today when I review with him, he said the 10kg is for over the year. So this round, he came back in 3 months, he managed to lose about 2kg. His sugar level dropped a little bit, lah. HbA1c is about 7 plus, which is ‘haven't reached the target’ but he do see a little bit of improvement. So, he say that he would do gradually.” (w2) | Positive weight loss and reduction in HbA1c |
| HP Foo | “I think it went quite well, because, uh, firstly the patient was very well prepared already, I mean he already saw his results, briefly went through you know the red and the green boxes [laughs], so he knew, and he also thought that his sugar wasn’t that well controlled initially, so he gave me like a very strict target, I mean, I think his level was like 7.8 or 7.9 and then his target was like 6.5 or below. So … after discussion with him - because I told him 6.5 might be a bit difficult in his circumstance, so I told him to taper down, maybe go to like 7 or near 7…. which I think in the second consult he did.” (w1) | Reduction in HbA1c |
| HP Wu | “One that went really well [involved a patient who had been prescribed cholesterol lowering medication but had agreed with a different doctor in a previous consultation that she would stop the medication to see if she could control it with the diet alone]. She signed on to be in the PACE-D program and she had her results sent to her and all that. And … she saw herself that her cholesterol results went up. And when she came back, it was even before I could talk to her about it, she said “Doctor, yeah, I like to talk to you to start back on the cholesterol medicine”. So, this was a decision that the patient arrived at on her own. And that’s probably because she had 2 weeks to think about this. She had her results at hand, and also a trending to see what it was like before and after, and she managed to put 2 and 2 together and was “OK, this was what my cholesterol was like on medication, this was what it was like without medication”. She made the connection, she made the decision. So… there was no effort on my part, really.” (w2) | (Patient requested) resumption of cholesterol-lowering medicine |
