# Supplementary material for: Implementing and evaluating care and support planning: a qualitative study of health professionals’ experiences in public polyclinics in Singapore
Source: BMC Prim Care. 2023 Oct 19;24:212. doi: 10.1186/s12875-023-02168-5 (PMC10585850; doi:10.1186/s12875-023-02168-5)
Supplement: Supplementary file 2 — Additional file 2. Sample Care Planning Letter (blank), English. [file 12875_2023_2168_MOESM2_ESM.pdf]

Goal Setting

What do you want to work on?

What do you want to achieve?

How important is it to you?

Not important 1 2 3 4 5 6 7 8 9 10 Important

Action Plan

What exactly are you going to do?

What might stop you and what can you do about it?

How confident do you feel?

Not confident 1 2 3 4 5 6 7 8 9 10 Confident

Review of Goals / Action Plan

Date: Time:

pace

National University Polyclinics
A member of the NUHS

REF.NO:9404

Diabetes Care Planning Results

Name: Mdm Tan	NRIC: XXXXX105I
Appointment Date: Thursday, March 7, 2019	Time: 11: 10 AM

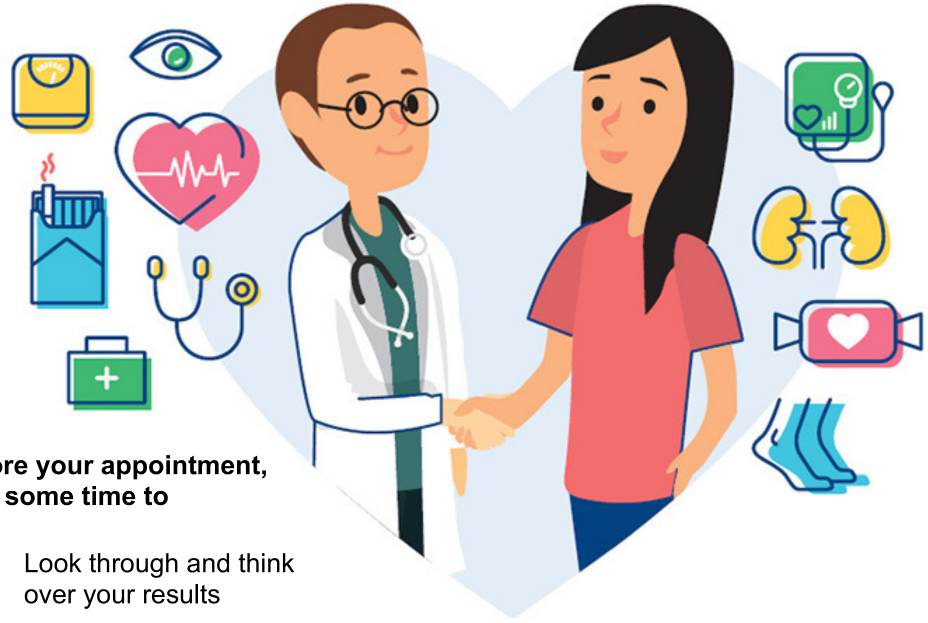

Before your appointment, take some time to

1. Look through and think over your results
2. Write down anything you would like to discuss with your doctor
3. Think about what you want to achieve and what you can do to achieve them

Please bring this to your appointment. We will use it to record the plan we make together about your diabetes treatment.

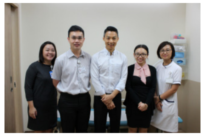

Dear Mdm Tan

What's important to you?
What aspects of your diabetes would you like to talk about? Write them down here.

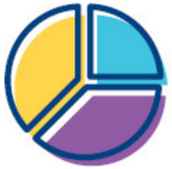

Issues of Concern

These are some issues that may be of concern for people living with diabetes.
Circle those that you wish to discuss with your doctor during the care planning consultation (You may circle more than one option).

Medical appointments	Work
Avoiding sugary food/drinks	Pregnancy & family planning
Healthier eating	Sexual health
Eating out	Monitoring glucose level
	Hypoglycemia (low glucose level)
Driving / Travel	
Giving up smoking	Medications
Alcohol	Foot care
Physical activities	Others

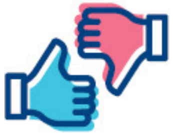

Your mood

Your mood is important.
In the past 2 weeks

- Have you been feeling down, depressed or hopeless?
- Have you had little interest or pleasure in doing things?

Your Annual Screening Checks

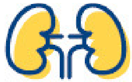

Kidney
Your kidneys are tested by looking at two tests:

1. A blood test (eGFR). The higher and more stable the eGFR, the better.

**eGFR**<br>Previous result:<br>101mL/min (27/03/18)	**Latest Result:**<br>**102mL/min (25/02/19)**<br>Normal: more than 60mL/min	Your risk:<br>Low

2. An early morning urine test to check the level of protein in your urine.

**Urine ACR**<br>Previous result:<br>35.5mg/g (13/12/18)<br>**Latest Result:**<br>**11.6mg/g (25/02/19)**<br>Normal: less than 30mg/g	**Urine PCR**<br>Previous result: -<br>**Latest Result:-**<br>Normal: less than 20mg/mmol	Your risk:<br>Low

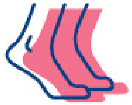

Feet
Your foot check detects if you have problems with blood supply or the feeling (sensation) in your feet.

Your Diabetic Foot Screening was last performed with us on 27 Mar 2018. Please book an appointment for the next session at the soonest date.	Last check:<br>27/03/18

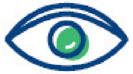

Eyes
Your eye check looks for any changes to tiny blood vessels at the back of your eye.

Your Diabetic Retinal Photography was last performed with us on 27 Mar 2018. Please book an appointment for the next session at the soonest date	Last check:<br>27/03/18

Your questions or thoughts:

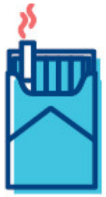

Smoking

Stopping smoking is one of the best things you can do to stay healthy.

Current smoking status:
**No**

Your questions or thoughts

Your risk:
**Low**

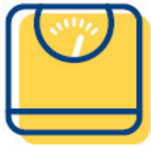

Weight

Being overweight increases the risk of many health problems. Maintaining a healthy weight helps with your blood glucose, cholesterol level and blood pressure control.

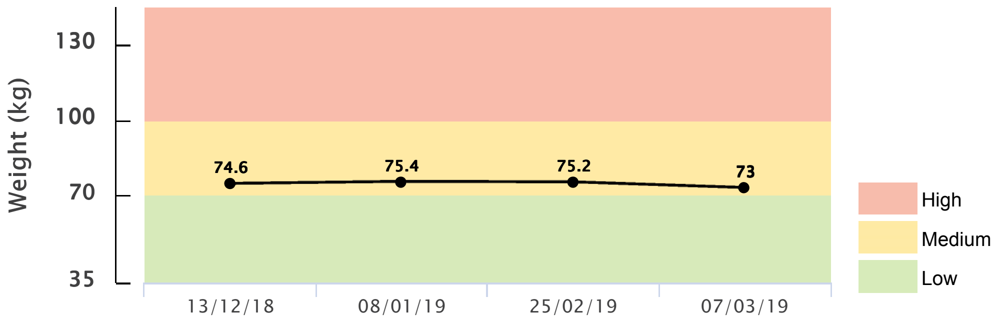

Your questions or thoughts

Your risk:
**Medium**

At a glance, let's look at how you are doing

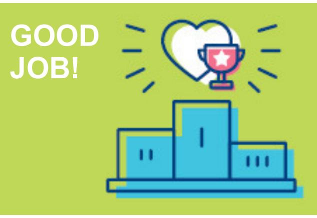

You have done well in these areas! Congratulations and keep it up!

- Blood Pressure
- Smoking Status
- Kidney

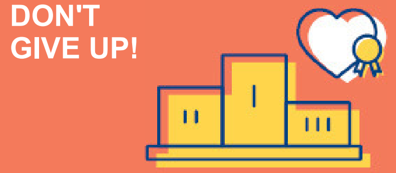

These areas may need attention or improvement. You may want to discuss them with your doctor.

- Blood Glucose Control (HbA1c)
- LDL Cholesterol
- Weight

Blood Glucose Control (HbA1c)

HbA1c tells you how your blood glucose has been over the last 8 to 12 weeks. Keeping your HbA1c on target will lower your risk of diabetes complications like blindness and kidney failure.

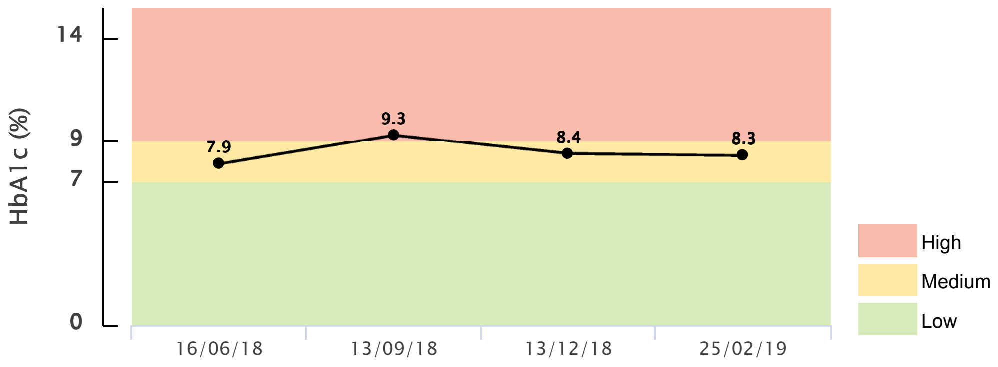

Your questions or thoughts:

Your risk:
Medium

LDL Cholesterol

This is a measure of bad cholesterol level in the blood. Maintaining a good level will reduce the risk of heart attack and stroke.

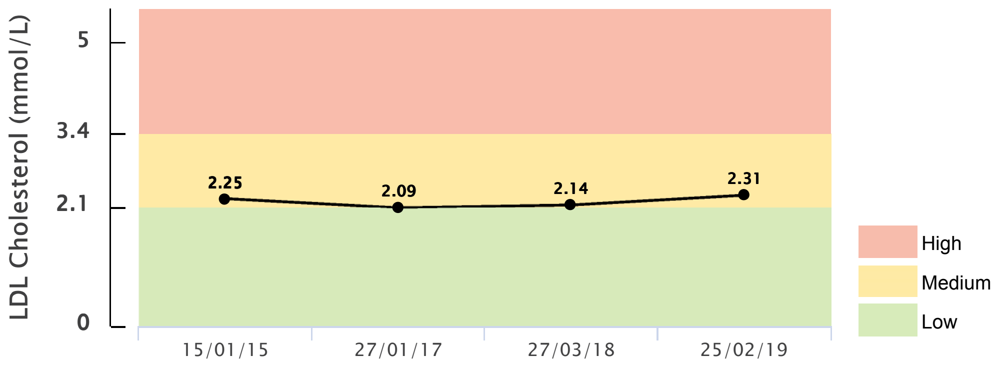

Your questions or thoughts:

Your risk:
Medium

Blood Pressure

Controlling your blood pressure will help to reduce the risk of heart attacks, strokes, kidney failure and blindness.

Systolic Blood Pressure (SBP)

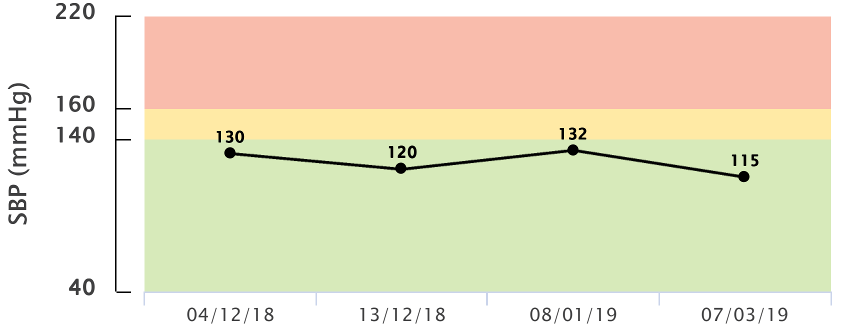

Diastolic Blood Pressure (DBP)

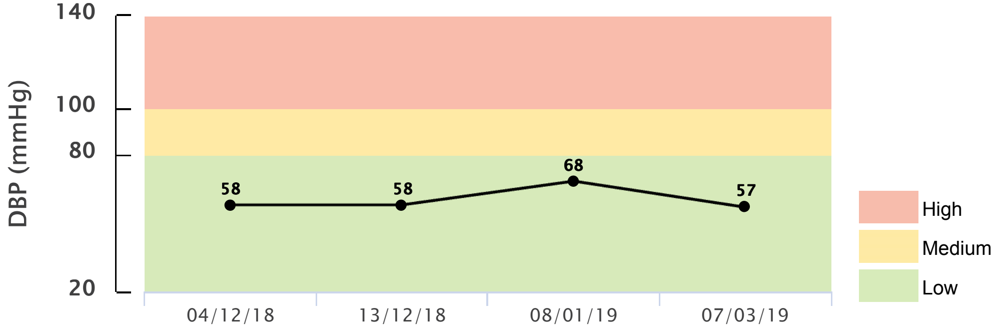

Your questions or thoughts

Your risk:
Low
